# Regional differences in epidermal growth factor receptor-tyrosine kinase inhibitor therapy in lung cancer treatment using a national database in Japan

**DOI:** 10.1038/s41598-023-31856-6

**Published:** 2023-03-30

**Authors:** Hiromi Matsumoto, Nobuaki Kobayashi, Satoru Shinoda, Atsushi Goto, Ayami Kaneko, Nobuhiko Fukuda, Chisato Kamimaki, Sousuke Kubo, Keisuke Watanabe, Nobuyuki Horita, Yu Hara, Yoshihiro Ishikawa, Takeshi Kaneko

**Affiliations:** 1 Department of Pulmonology, Yokohama City University Graduate School of Medicine, 3-9 Fukuura, Kanazawa-ku, Yokohama, 236-0004 Japan; 2 Department of Biostatistics, Yokohama City University Graduate School of Medicine, 3-9 Fukuura, Kanazawa-ku, Yokohama, Japan; 3 Department of Health Data Science, Yokohama City University Graduate School of Medicine, 3-9 Fukuura, Kanazawa-ku, Yokohama, Japan; 4 Respiratory Disease Center, Yokohama City University Medical Center, 4-27 Urahunecho, Minami-ku, Yokohama, Japan; 5 Department of Surgery, Yokohama City University Graduate School of Medicine, 3-9 Fukuura, Kanazawa-ku, Yokohama, Japan

**Keywords:** Cancer, Cancer epidemiology, Lung cancer

## Abstract

Epidermal growth factor receptor-tyrosine kinase inhibitors (EGFR-TKIs) are extensively used in the treatment of non-small cell lung cancer (NSCLC); hence, equal access to them is important. Therefore, this study aimed to identify regional differences in the prescription of EGFR-TKIs and the factors contributing to these differences. In this ecological study, we collected data using the National Database Open Data and the National Cancer Registry. The standardized claim ratio (SCR) was used as an indicator of the number of EGFR-TKI prescriptions. Additionally, we examined the association between SCR and various factors to identify the factors associated with this difference. The average SCR for the top three provinces was 153.4, while the average for the bottom three provinces was 61.6. Multivariate analysis used for evaluating the association of SCR with variables revealed that the number of designated cancer hospitals and radiation therapies were independent factors associated with the SCR of EGFR-TKIs. There were significant regional differences in the prescriptions of EGFR-TKIs in Japan based on the number of coordinated designated cancer hospitals and the number of patients receiving radiotherapy alone. These findings emphasize the need to implement policies to increase the number of hospitals to reduce regional differences.

## Introduction

Japan has a universal health insurance system which provides equal access to health care. Under the insurance system, there is no difference in the cost of the same treatment between hospitals. Additionally, several measures have been taken to reduce the uneven distribution of doctors and the per capita cost of healthcare in different regions^1,2^. Furthermore, the Basic Law on Cancer Control was implemented in Japan in April 2007 to ensure uniform access for cancer patients to treatment irrespective of their place of residence. However, there are no reports on differences in lung cancer treatment between prefectures in Japan; therefore, the ground reality is unclear. Myrdal et al. reported that the treatment of lung cancer in Sweden varied from county to county^3^. Regional differences in the prescription of other medications have also been reported in Japan^4,5^. Therefore, it can be assumed that regional differences exist in cancer treatment in Japan, and should be verified.

EGFR-TKIs have demonstrated favorable progression-free survival and overall survival compared to cytotoxic agents in the treatment of NSCLC with EGFR mutations^6,7^. Therefore, it is now the first-choice treatment as per national and international guidelines^8,9^, and if indicated, it should be available to as many patients as possible.

In Japan, the National Database (NDB) of Health Insurance Claims and Specific Health Checkups of Japan was established in 2009. The NDB contains information on medical statements prepared by medical institutions to claim reimbursement from insurers. Moreover, since 2016, the basic aggregate table has been created and made available to the public as open data^10^.

Therefore, this study aimed to identify regional differences in the prescription of EGFR-TKIs and the associated factors using NDB open data.

## Results

### Number of lung cancer patients prescribed EGFR-TKIs in each prefecture in Japan

Table 1 shows the number of patients, the number of prescribed drugs, and the number of EGFR gene tests per patient in each prefecture. Gefitinib and osimertinib were the most frequently prescribed EGFR-TKIs in most prefectures. The prefectures with the highest and lowest numbers were Tokyo and Tottori with 11,702 and 642 patients respectively. The median number of patients was 1701. The morbidity rate per 100,000 people was 103.0 (range: 66.9–125.7). Gefitinib, an EGFR-TKI, became available in 2005 in Japan^11^. Osimertinib is the recommended first line of treatment for patients with sensitive EGFR mutation based on domestic and international guidelines for advanced NSCLC^8,9^.

**Table 1:** Numbers of patients and prescribed drugs in each prefecture.

Prefecture	Number of patients (2018)	Number of prescriptions (2018)				EGFR test (per patient)
		Gefitinib	Erlotinib	Afatinib	Osimertinib	
Hokkaido	6582	76,649	26,742	36,432	55,778	0.3645
Aomori	1366	14,820	8146	14,093	12,408	0.5300
Iwate	1148	22,299	7239	11,113	14,709	0.4791
Miyagi	2205	25,803	7020	14,941	17,411	0.5311
Akita	1104	19,673	5111	7120	15,474	0.5308
Yamagata	1147	16,453	NA	7817	10,264	0.4464
Fukushima	1788	21,079	9294	13,072	15,842	0.4290
Ibaraki	2717	24,043	9041	18,672	22,216	0.2797
Tochigi	1701	18,306	4343	15,366	15,635	0.6173
Gunma	1813	18,835	3958	16,848	17,412	0.3861
Saitama	6256	62,835	20,121	40,201	56,403	0.4004
Chiba	5563	88,302	23,426	32,553	73,152	0.3825
Tokyo	11,702	153,938	62,755	75,332	107,237	0.4963
Kanagawa	7646	95,514	37,738	69,661	80,317	0.4541
Niigata	2395	30,390	7328	14,002	19,088	0.4058
Toyama	1101	17,064	2852	5217	9597	0.4877
Ishikawa	1286	10,343	8330	11,666	10,885	0.4705
Fukui	786	4193	5779	6772	6390	0.7277
Yamanashi	759	7541	1481	3682	7909	0.4071
Nagano	1836	34,203	10,925	18,662	20,027	0.3764
Gifu	1986	17,278	12,574	10,644	13,157	0.4668
Shizuoka	3376	26,647	11,586	12,951	20,650	0.4399
Aichi	6440	71,246	30,497	42,164	61,591	0.6081
Mie	1861	24,203	4601	12,948	21,814	0.2853
Shiga	1414	17,982	7214	12,264	13,683	0.3692
Kyoto	2726	38,055	18,677	22,801	28,396	0.4938
Osaka	9270	97,862	38,720	72,189	96,472	0.4787
Hyogo	5651	55,668	29,571	68,639	66,030	0.4574
Nara	1484	8703	3509	2678	8411	0.4468
Wakayama	1157	18,525	4584	7497	12,853	0.5169
Tottori	642	10,518	NA	7325	10,666	0.5047
Shimane	763	12,403	3864	7085	9480	0.3526
Okayama	1895	9534	5080	11,455	13,395	0.5245
Hiroshima	2839	29,600	15,096	27,016	22,474	0.5280
Yamaguchi	1580	18,220	6386	15,787	10,830	0.4411
Tokushima	800	10,692	5644	7660	8776	0.3775
Kagawa	1107	13,387	12,658	5416	11,252	0.4381
Ehime	1602	15,776	3506	11,454	9696	0.3814
Kochi	765	8056	9699	4214	7777	0.4797
Fukuoka	5291	54,640	30,719	37,943	57,244	0.4339
Saga	877	11,031	3955	8067	4797	0.4675
Nagasaki	1685	21,155	5147	10,731	14,456	0.3128
Kumamoto	1705	12,475	9664	6824	10,109	0.4669
Oita	1194	16,197	10,907	8599	7909	0.4933
Miyazaki	1125	14,045	4794	7350	10,754	0.4080
Kagoshima	1719	19,448	9667	15,225	15,061	0.3589
Okinawa	970	12,016	2303	7289	8431	0.3392

*EGFR* Epithelial growth factor receptor.

### Regional differences in prescription of EGFR-TKI

**Figure 1:**
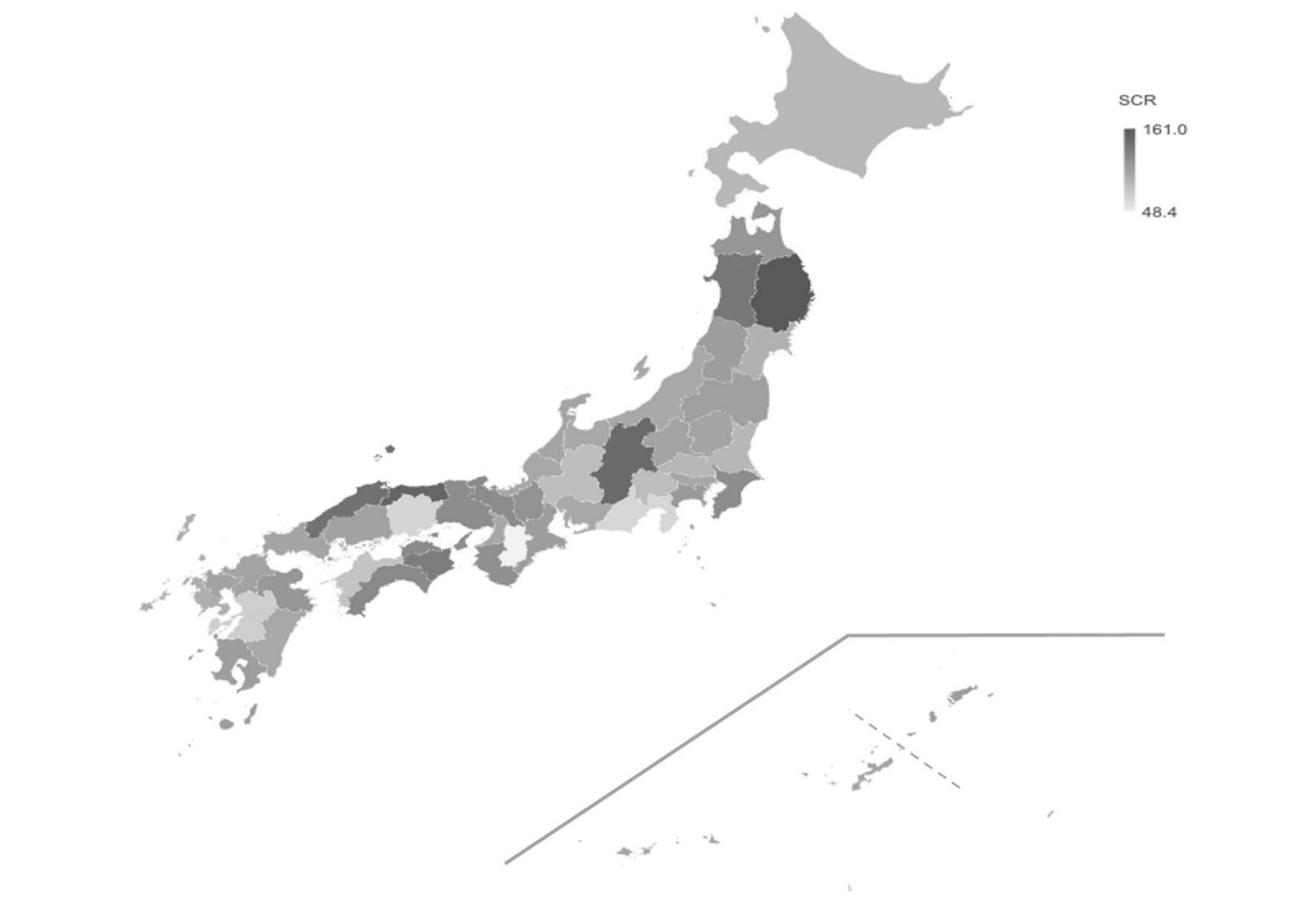
Intensity map showing SCR for each prefecture. The darker the color, the larger the SCR. A part of Kagoshima prefecture and Okinawa prefecture (the lower right of the figure) is different from the actual position.

The SCRs for each prefecture from 2016–2018 are shown in Supplementary Table S1, and the SCRs for 2018 are illustrated on the map in Fig. 1. The median SCR for 2018 was 104.2, with a range of 48.4–161.0. Three prefectures with the highest SCR were Iwate (161.0), Tottori (151.7), and Nagano (147.6) in 2018. Three prefectures with the lowest SCR were Nara (48.4), Shizuoka (67.7), and Okayama (68.9) in 2018. The top three prefectures were also in the top six prefectures in 2017 and 2016. The bottom three prefectures were all also in the bottom five prefectures in 2017 and 2016. In 2018, the average SCR for the top three prefectures was 153.4, while the average for the bottom three prefectures was 61.6.

### Univariate analysis of factors related to the prescription of EGFR-TKIs

**Figure 2:**
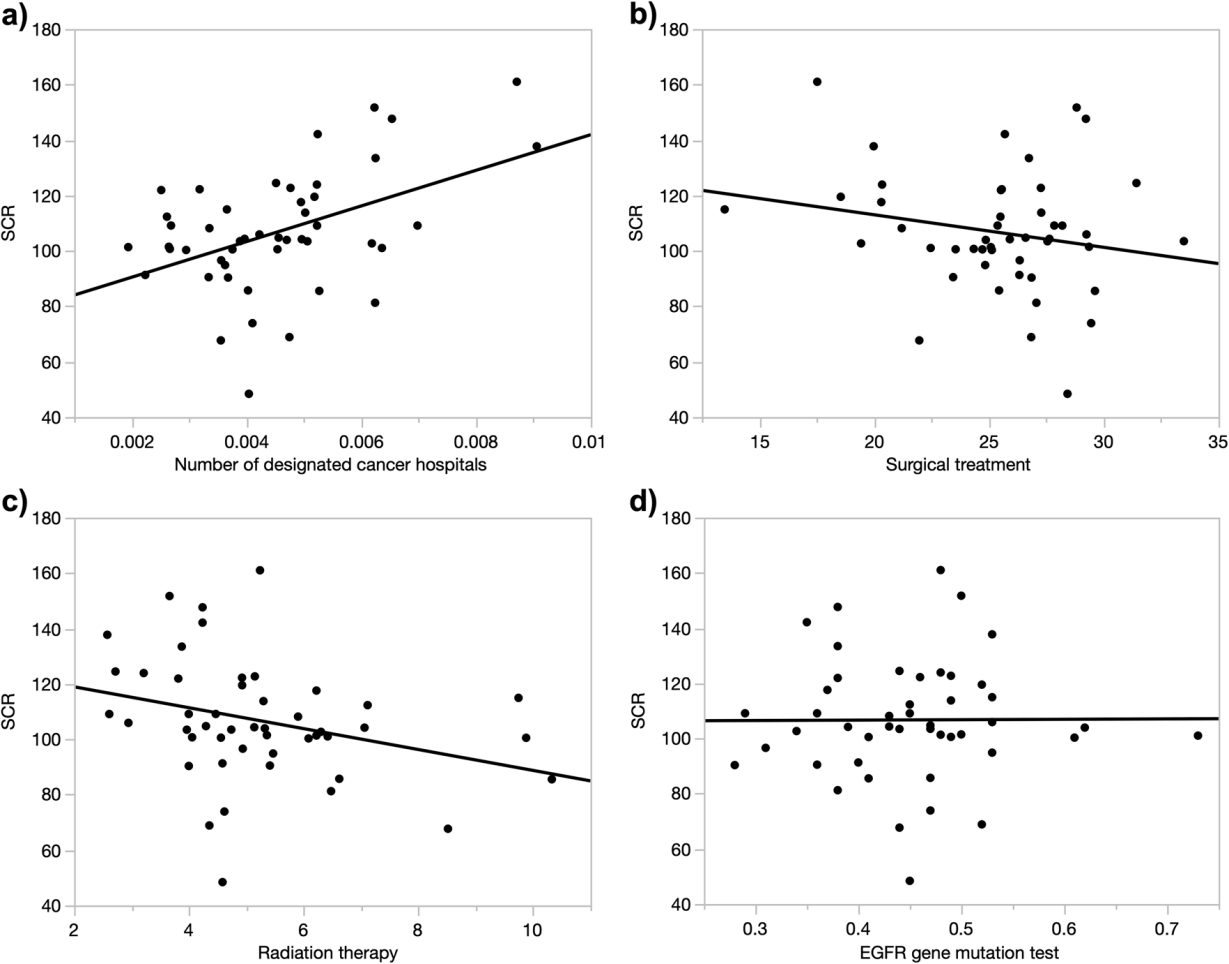
Scatter plot with the value of each explanatory variable on the x-axis and SCR on the y-axis. (**a**) The explanatory variable is the number of designated cancer hospitals. y = 77.58 + 6434.4x, *R*^2^ = 0.21. (**b**) The explanatory variable is the proportion of surgical treatment. y = 136.5 − 1.175x, *R*^2^ = 0.042. (**c**) The explanatory variable is the proportion of radiation therapy. y = 126.5 − 3.777x, *R*^2^ = 0.094. (**d**) The explanatory variable is the number of EGFR gene mutation test per lung cancer patient. y = 106.0 + 1.492x, *R*^2^ = 0.00003. *R*^2^: coefficient of determination; SCR: standardized claim ratio.

Pearson’s correlation coefficients between SCR and each factor are shown in Table 2. Weak correlations were found for the number of designated cancer hospitals per lung cancer patient (*r* = 0.46, *p* = 0.0012, Fig. 2a), the number of respiratory surgery specialists per lung cancer patient (*r* = 0.32, *p* = 0.03, Figure not shown), the percentage of patients treated with surgery as initial treatment (*r* = − 0.21, *p* = 0.17, Fig. 2b), the percentage of patients treated with radiation alone (*r* = − 0.31, *p* = 0.04, Fig. 2c), and the percentage of patients treated with surgery plus drug therapy (*r* = 0.28, *p* = 0.06, Figure not shown). Of these, regression coefficients were significant for the number of designated cancer hospitals per lung cancer patient, the number of respiratory surgery specialists per lung cancer patient, and the percentage of patients receiving only radiotherapy as initial treatment. In addition, there was no correlation between the number of EGFR tests per lung cancer patient and SCR (*r* = 0.007, *p* = 0.96, Fig. 2d). Neither gender nor smoking history was correlated with SCR (*r* = 0.018, *p* = 0.90; *r* = − 0.034, *p* = 0.82, respectively, Table 2), although these are factors that are clearly associated with the positivity of EGFR mutations. The scatter plots of these four factors and SCR are shown in Fig. 2.

**Table 2:** Correlation between SCR and each factor in 2018.

	*r*	*p*
Characteristics of people and patients in each prefecture		
Percentage of LC patients over 80 years old	0.19	0.19
Percentage of LC female patients	0.018	0.90
Smoking rate for people 40 years and older	− 0.034	0.82
Medical resources (number per lung cancer patient)		
Respiratory specialists	− 0.15	0.32
Respiratory Surgery Specialist	0.32	0.027*
Cancer pharmacotherapy specialists	− 0.035	0.82
Designated cancer hospitals	0.46	0.0012*
How it was found (percentage of each to diagnosed cases)		
Cancer screening	0.14	0.35
During follow-up of other diseases	− 0.054	0.72
Progress at diagnosis (percentage of each to diagnosed cases)		
Local	− 0.15	0.32
Lymph node metastasis	0.015	0.92
I nvasion to adjacent organs	0.13	0.37
Metastasis	0.044	0.77
Initial treatment (percentage of each to diagnosed cases)		
Surgical treatment	− 0.21	0.17
Radiation therapy	− 0.31	0.036*
Pharmacotherapy	0.078	0.60
Pharmacotherapy + Radiation therapy	0.028	0.85
Surgical treatment + Radiation therapy	− 0.071	0.64
Surgical treatment + Pharmacotherapy	0.28	0.057
Surgical treatment + Pharmacotherapy + Radiation therapy	− 0.15	0.33
Genetic test (number of cases per lung cancer patient)		
EGFR gene mutation test	0.0070	0.96
ROS1 fusion gene test	0.042	0.78

*r* Pearson’s correlation coefficient, * *p* < 0.05, *SCR* Standardized claim ratio, *EGFR* Epithelial growth factor receptor, *ROS1* c-ros oncogene 1.

### Factors Correlating with Regional Differences in EGFR Testing Rates

Regional differences on the prevalence of EGFR gene mutations and the type of gene mutation (e.g., exon 19 deletion or L858R) were not available. Therefore, we examined patient characteristics associated with positivity for the EGFR gene mutation. The distribution of the percentage of lung cancer patients aged 80 years or older for each prefecture is shown in Table 3. The median value was 33.4 (range 27.1–37.8, standard deviation (SD) 2.66). The proportion of female patients was 32.6 (range 28.2–36.2, SD 2.02), the proportion of smokers over 40 years old was 29.3 (range 24.2–40.3, SD 3.18).

**Table 3:** Distribution of factors associated with EGFR gene mutations.

	Minimum	Median	Maximum	SD
Percentage of LC patients over 80 years old	27.1	33.4	37.8	2.66
Percentage of LC female patients	28.2	32.6	36.2	2.02
Smoking rate (%)	24.2	29.3	40.3	3.18
Surgical treatment (%)	13.5	25.7	33.5	3.79
Radiation therapy (%)	2.58	4.93	10.33	1.76

*LC* Lung cancer, *SD* Standard deviation.

As for regional differences in treatment, the proportion of patients whose initial treatment for lung cancer was surgery was 25.7 (range 13.5–33 0.5, SD 3.79) and 4.93 (range 2.58–10.33, SD 1.76) for first-line treatment with radiation alone. Both factors showed less regional variation by prefecture compared to the SCR for EGFR-TKIs (range 48.4–161, SD 21.7).

**Figure 3:**
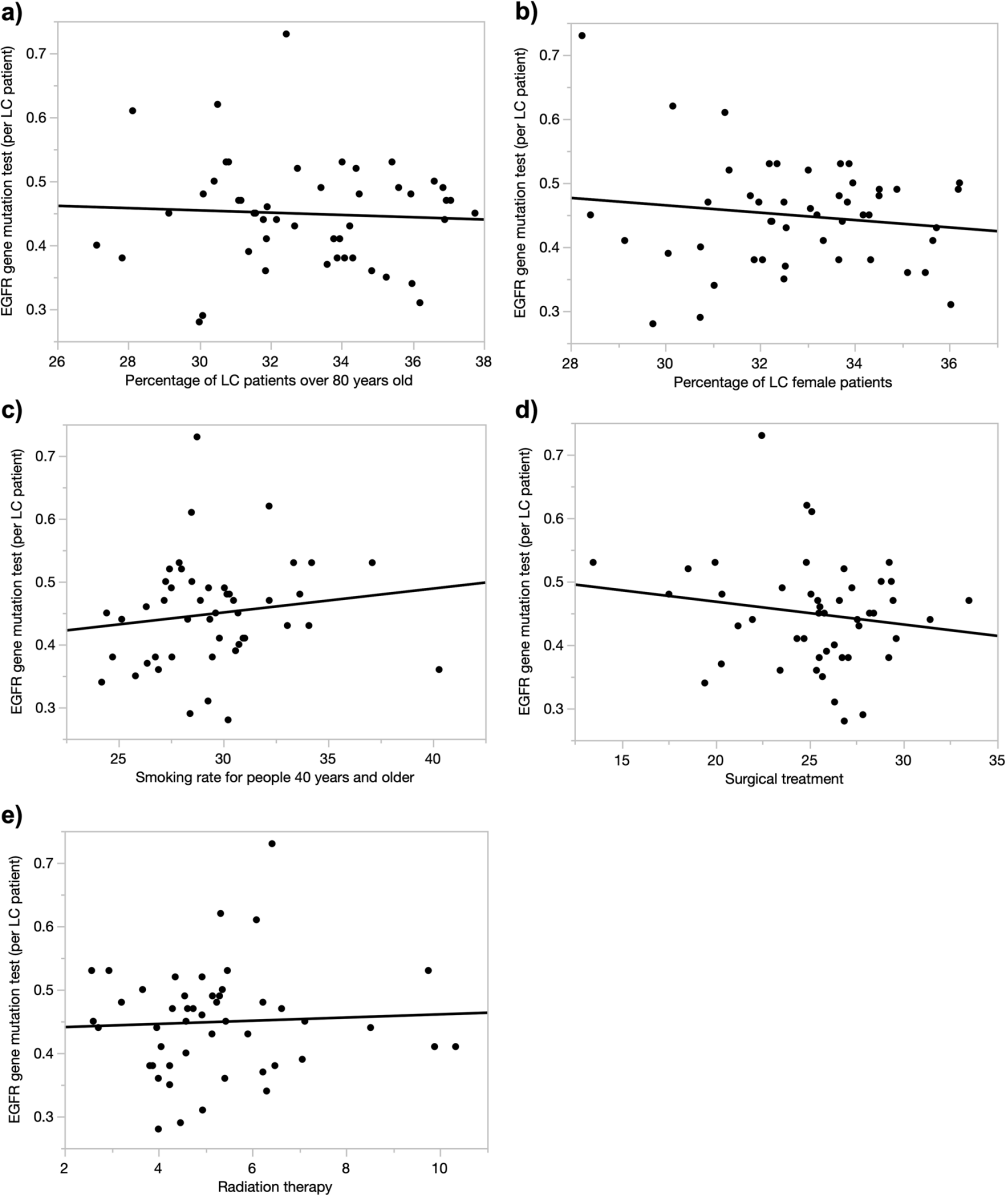
Scatter plots with the value of each explanatory variable on the x-axis and the number of EGFR gene tests performed per lung cancer patient on the y-axis. (**a**) The explanatory variable is the percentage of LC patients over 80 years old. y = 75.3 + 102.9x, *R*^2^ = 0.0031. (**b**) The explanatory variable is the percentage of LC female patients. y = 0.64  − 0.0058x, *R*^2^ = 0.019. (**c**) The explanatory variable is the smoking rate for people 40 years and older. y = 0.34 − 0.0038x, *R*^2^ = 0.020. (**d**) The explanatory variable is the percentage of surgical treatment. y = 0.54 + 0.0036x, *R*^2^ = 0.026. (**e**) The explanatory variable is the percentage of radiation therapy. y = 0.44 + 0.0025x, *R*^2^ = 0.0027. *R*^2^: coefficient of determination.

We examined the correlation between these factors and EGFR gene mutation testing, but no correlation was found (Fig. 3).

### Multivariate analysis of factors related to EGFR-TKI prescription

To identify factors related to differences in regional SCRs, four clinically relevant variables were selected for multiple regression analysis. The results are shown in Table 4, and the number of designated cancer hospitals and radiation therapy were identified as independent factors. The standardized partial regression coefficient showed that the item with the strongest effect was the number of designated cancer hospitals. Multivariate analysis also showed no association between EGFR gene mutation test and SCR.

**Table 4:** Multiple regression analysis with SCR in 2018 as the objective variable.

Explanatory variables	B (95% CI)	*p*	*β*	VIF	*R*^2^
Number of designated cancer hospitals (per LC patient)	4981.1 (1059.6 to 8902.7)	0.0140*	0.355	1.17	0.302
Surgical treatment (%)	− 1.32 (− 2.97 to 0.33)	0.115	− 0.230	1.41	
Radiation therapy (%)	− 3.73 (− 7.24 to − 0.22)	0.0376*	− 0.303	1.22	
EGFR gene mutation test (per LC patient)	− 14.79 (− 82.28 to 52.71)	0.661	− 0.0580	1.15	

*B* Partial regression coefficient, *β* Standardized partial regression coefficient, *VIF* Variance inflation factor, *R*^*2*^ Coefficient of determination, * p < 0.05, *SCR* Standardized claim ratio, *95% CI* 95% confidence interval, *EGFR* Epithelial growth factor receptor; *LC* Lung cancer.

## Discussion

In this study, we analyzed the regional difference in SCR of EFGR-TKIs, and the related factors using NDB database. The SCR average of EGFR-TKIs was approximately three times higher in the high SCR region than that in the low SCR region (range: 48.4–161.0) (Fig. 1 and Supplementary Table S1). Similar studies using Japanese SCR have been reported. There were significant regional differences (regions with the highest prescription rate had a 1.8-fold SCR as compared to that of the regions with the lowest prescription rate) in the SCRs of antipsychotics (range: 67.9–122.8)^12^. In addition, a study on regional differences in polypharmacy found that the regions with the highest prescription had a 1.9-fold SCR compared to that of the regions with the lowest prescription (range: 76.1–142.6)^13^. The indication of EGFR-TKIs is limited to EGFR mutation-positive patients diagnosed with advanced NSCLC. Therefore, regional differences in SCR are less likely to occur compared to the latter two studies.

The number of designated cancer hospitals per patient was positively related to SCR of EGFR-TKIs. The percentage of patients who received monotherapy with radiation as initial treatment negatively correlated with SCR. However, the number of EGFR gene mutation testing per patient is not associated with SCR. Factors associated with regional differences in EGFR-TKI prescriptions might contribute to improving the treatment of lung cancer patients.

We identified that the percentage of patients treated with radiation alone was significantly associated with SCR of EGFR-TKIs (*b* = − 3.77, *r* = − 0.31, *p* = 0.04, Fig. 2c). The percentage of patients treated with surgery and chemotherapy was also weakly correlated with SCR (*b* = 3.99, *r* = 0.28, *p* = 0.06, Figure not shown). Patients who received radiation alone were generally those who could not tolerate surgery or chemotherapy due to old age, poor performance status, or other reasons^8,9^. Regions with more patients treated with radiation alone had lower SCR of EGFR-TKIs, as these patients are generally not treated with EGFR-TKIs.

Another factor significantly related to SCR of EGFR-TKIs was the number of designated cancer hospitals per lung cancer patient (*b* = 6434.4, *r* = 0.46, *p* = 0.00, Fig. 2a). There are various requirements for certification as a designated cancer hospital in Japan, such as full-time staff including medical oncologists, surgeons, radiologists, pharmacists, and nurses dedicated to the outpatient chemotherapy unit^14^. Several studies showed better survival of patients with NSCLC in stage I-IIIA treated at designated cancer hospitals than that at community hospitals^15–18^. Ramalingam et al.^19^ reported that the overall survival of NSCLC patients treated in academic centers was significantly longer than that in community centers. This is mainly associated with the progress in treatments for patients with adenocarcinoma harboring driver mutation^20^. As discussed below, the number of EGFR tests per patients is not related to the SCR of EGFR-TKIs (r = 0.0070, Fig. 2d). Therefore, the reason for the close relationship between the number of designated cancer hospitals and the SCR of EGFR-TKIs might be the high positive rate in EGFR testing. There are various reasons for differences in indications for EGFR testing at each hospital. The positive pretest rate in patients with squamous cell carcinoma or small cell carcinoma is lower than that in adenocarcinoma^21^. Moreover, the accuracy of EGFR testing may be different in those hospitals. Several studies indicated the discrepancy in results of EGFR testing by Polymerase chain reaction (PCR), Next-Generation sequencing (NGS), tumor tissue, and liquid biopsy^22–24^. If the number of false-negative EGFR mutation tests are reduced, the number of EGFR-TKI prescriptions will increase, and the SCR will also increase.

In the present study, no correlation was observed between EGFR mutation test and SCR (*r* = 0.0070, Fig. 2d). EGFR testing rate has a significant impact on the treatment of NSCLC patients. EGFR testing was associated with longer overall survival. Hence, EGFR testing increased overall survival among the patients with EGFR mutation treated with EGFR-TKIs^25^. The reported EGFR testing rates were 42.5, 27.0, 64.8, 33.5, and 54.5% in north China, New Zealand, Japan, Korea, and Taiwan, respectively^25–27^. EGFR testing is more frequently performed in areas with high health insurance coverage, areas with cancer centers, and urban areas^26^. Our data suggest that EGFR testing is not related to prescribing EGFR-TKIs in Japan. The difference in the indication of EGFR testing may be the reason for this discrepancy because Japan has a relatively uniform social security system.

In this study, we did not obtain prefecture-specific information on EGFR positivity rates or histology. Therefore, we examined factors associated with EGFR mutations, including adenocarcinoma, sex, and nonsmokers^28^. We examined the proportion of women with lung cancer and the smoking rate in each prefecture for those over 40 years of age, and found little regional difference compared to the SCR.

To investigate the possibility that EGFR testing is not performed due to sex, age, or clinical stage, univariate analysis was performed for EGFR testing in lung cancer patients and the proportion of female and older patients, smoking rates, and first-line treatment (surgery or radiotherapy), but no correlation was found for any of these factors. This suggests that at the prefectural level, there is no selection of patients for EGFR mutation testing based on patient background or other factors.

A large value of SCR is not necessarily desirable due to the possibility of over-prescription. One of the reasons for prescribing more EGFR-TKIs than necessary may be due to differences in the standards adopted. For example, with respect to afatinib, there are four standard doses available in Japan: 20, 30, 40, and 50 mg^29^. Since the SCR is calculated based on the number of tablets prescribed, the different prescription standards can contribute to differences in SCR. The SCR will also be higher if EGFR-TKIs are used continuously after the disease has progressed or if they are used in cases where they are not strictly indicated.

## Limitations

This study had several limitations. First, the SCR may not be accurate because of the unpublished data in the NDB open data. In particular, the data for provinces with small SCRs might have been overestimated. Second, the study did not provide data on regional differences in the histology or EGFR mutation prevalence and did not adjust for these effects. Regional differences in the trends in histology and mutation may be associated with SCR; however, the magnitude of this effect is unknown. The NDB data does not contain clinical information, including the histological subtype of lung cancer and the treatment line of EGFR-TKI. EGFR-TKIs are only useful for patients with EGFR mutation. EGFR gene mutations are more common in adenocarcinoma^8,9^. Moreover, osimertinib, which is a third-generation TKI, may be used as the first-line treatment as well as the second-line or later. Third, the sensitivity of EGFR gene mutation detection varies depending on the testing method^30,31^, and differences in specimen processing methods and fixation time may affect the results^32,33^. However, in this study, these prefecture-specific information could not be obtained, which could lead to bias. Fourth, the number of EGFR-TKI prescriptions was based on the location of the hospital. Moreover, the number of patients in each prefecture was calculated based on the patients’ registered addresses. Hence, we could not modify the effect of patients who underwent treatment in a different prefecture. Finally, this study used only population data, and the present results may not be applicable at the individual level.

Nevertheless, the present study is the first to examine regional differences in lung cancer treatment using NDB open data, which includes data from almost all insurance practices. Further validation is needed to identify regional differences in cancer treatment and the factors influencing them to reduce these disparities.

## Conclusion

Regional differences exist in the propensity to prescribe EGFR-TKIs for lung cancer. Furthermore, the tendency to prescribe EGFR-TKIs increased with the number of coordinated designated cancer hospitals and decreased with the number of patients receiving radiotherapy alone. Therefore, in areas with few coordinated designated cancer hospitals, efforts to increase the number of those hospitals may reduce these regional differences.

## Methods

### National health insurance system and NDB open data

In Japan, medical care is covered by a public health insurance system and the cost incurred by the patient depends on their income^34^. The remaining amount is billed by each medical institution to the Claims Review and Reimbursement Organizations located in each of the 47 prefectures and paid if the claim is found to be appropriate^35^. Medical fees for consultations, examinations, and treatment are standardized throughout Japan, and each medical institution must provide medical care at this fixed price.

The NDB was established in 2009 in accordance with the "Act on Assurance of Medical Care for the Elderly People." It is one of the largest databases in the world, accumulating information on receipts since 2009 and information on specific health checkups and specific health guidance since 2008. As part of efforts to promote the utilization of these data, the actual state of medical care in Japan and the results of specific health checkups were presented as statistical data for the first time as NDB open data in 2016. The NDB open data consists of seven major categories: "Medical Practice," "Dental Practice," "Dental Injuries and Diseases," "Drugs," "Specific Health Care Materials," "Specific Health Examination (Laboratory Test Values)," and "Specific Health Examination," and the tabulated results are freely available to anyone. The tabulated results are prepared by fiscal year, and as of 2022, data up to the fiscal year 2019 are available^10,36,37^.

### Study design

This study adopted an ecological study design at the prefecture level. Using data on the number of prescriptions for oral medications generated from almost all insurance data, we examined differences in the propensity to prescribe EGFR-TKIs between prefectures. All the clinically available EGFR-TKIs are delivered orally. We also examined the impact of differences in medical resources (number of specialists and hospitals) and conditions at diagnosis in each prefecture on prescribing trends.

### Data sources

Using the third, fourth, and fifth NDB open data, we obtained the number of EGFR-TKI (gefitinib, erlotinib, afatinib, osimertinib) prescriptions (number of tablets and capsules) for outpatient prescriptions in each prefecture, the number of EGFR-TKI prescriptions by sex and age group nationwide, and the number of EGFR gene mutation tests and ROS1 fusion gene tests in each prefecture. Data on the number of in-hospital prescriptions of EGFR-TKIs were not available for this study due to NDB rules (data from prefectures with low numbers of prescriptions are not published) and therefore were not included. We also obtained data on smoking rates for those aged 40 and older in each prefecture from the Specific Health Examinations section of the NDB Open Data. The national cancer registry (2016–2018, https://www.e-stat.go.jp/stat-search/files?page=1&toukei=00450173&tstat=000001133323) was used to obtain information on the number of lung cancer patients by sex and age group, the circumstances of cancer detection, the degree of progression, and the nature of initial treatment in each prefecture in Japan. In addition, we obtained the number of respiratory specialists from the Japanese Respiratory Society website, the number of cancer pharmacotherapy specialists from the Japanese Society of Medical Oncology website, the number of respiratory surgery specialists from the Japanese Board of General Thoracic Surgery website, and the number of designated cancer hospitals from the Ministry of Health, Labor, and Welfare website, all in 2021.

### Indicators

As an indicator of the number of EGFR-TKI prescriptions, the standardized claim ratio (SCR) was calculated using the following formula^38^.

$$ SCR = \frac{actual\;number\;of\;prescriptions}{{expected\;number\;of\;prescriptions}} \times 100 $$

$$ Expected\;number\,of\;prescriptions = \sum \frac{A \times B}{C} $$

*A* = *number of lung cancer patients in eac*ℎ *prefecture by age and sex*, *B* = *number of prescriptions in Japan by age and sex*, *C* = *lung cancer patients by age and sex in Japan.*

The SCR is used to adjust for differences in the age and sex composition of each prefecture, and a score of 100 or more indicates that the number of cases is higher than the national average.

We also examined the association between SCR and the number of respiratory specialists, cancer pharmacotherapy specialists, designated cancer hospitals, how the disease was discovered, the degree of progression at diagnosis, the nature of the first treatment, the number of EGFR gene mutation tests, the number of ROS1 fusion gene tests, the percentage of older patients, the percentage of female patients, and the percentage of smokers over 40 years old in each prefecture in 2018. Although there are seven categories of designated cancer hospitals, the total number of such hospitals in each prefecture was used in this study. The number of medical specialists, the number of designated cancer hospitals, and the number of genetic tests performed per lung cancer patient in each prefecture were also used for analysis.

In the NDB open data, the number of outpatient prescriptions of oral medications was calculated using a larger estimate of 1,000 prescriptions for the prefectures where the number of prescriptions was less than 1,000 because the figures for those prefectures were not disclosed.

### Statistical analysis

JMP Pro 16 software (SAS Institute Inc.) was used for statistical analysis. To examine differences in prescription values, descriptive statistics were used to tabulate the characteristics of each prefecture. To examine the relationship between SCR and clinically important factors, multiple regression analysis was used. Pearson's correlation coefficient (*r*) and regression coefficient (*b*) were also calculated as supplementary analyses. No correlation was determined when $\left|r\right|$ < 0.2, weak correlation when 0.2 ≤ $\left|r\right|$  < 0.4, correlation when 0.4 ≤ $\left|r\right|$  < 0.7, and strong correlation when 0.7 ≤ $\left| r \right|$^39^. In each case, the significance level was set at 5%.

### Ethical considerations

This study was approved by the Institutional Review Board of the Yokohama City University. The NDB open data are anonymized medical claims data compiled and released by the Japanese Ministry of Health, Labor, and Welfare; thus, the information is de-identified. Therefore, the need for informed consent was waived.

## Supplementary Information

Supplementary Information.

## Data Availability

The data used in this study was obtained from the following websites: NDB open data: https://www.mhlw.go.jp/stf/seisakunitsuite/bunya/0000177182.html. National Cancer Registry: https://www.e-stat.go.jp/stat-search/files?page=1&toukei=00450173&tstat=000001133323.
