# Supplementary material for: Regional differences in epidermal growth factor receptor-tyrosine kinase inhibitor therapy in lung cancer treatment using a national database in Japan
Source: Sci Rep. 2023 Mar 30;13:5208. doi: 10.1038/s41598-023-31856-6 (PMC10063675; doi:10.1038/s41598-023-31856-6)
Supplement: Supplementary file 1 — Supplementary Information. [file 41598_2023_31856_MOESM1_ESM.docx]

**Supplemental Table S1. Year-wise SCRs for each prefecture**

| **Prefecture** | **SCR** | | |
| --- | --- | --- | --- |
|  | **2016** | **2017** | **2018** |
| Hokkaido | 99.0 | 93.6 | 90.5 |
| Aomori | 109.8 | 107.7 | 114.9 |
| Iwate | 158.7 | 138.4 | 161 |
| Miyagi | 104 | 89.1 | 94.9 |
| Akita | 156.3 | 153.7 | 137.7 |
| Yamagata | 133.1 | 95.9 | 109.1 |
| Fukushima | 110.6 | 106.9 | 108.2 |
| Ibaraki | 91.7 | 87.0 | 90.3 |
| Tochigi | 111.7 | 93.7 | 103.9 |
| Gunma | 114.6 | 90.1 | 104.2 |
| Saitama | 80.9 | 87.5 | 91.3 |
| Chiba | 118.6 | 128.0 | 121.9 |
| Tokyo | 102.7 | 102.5 | 101.5 |
| Kanagawa | 115.2 | 105.9 | 112.3 |
| Niigata | 81.2 | 90.7 | 100.5 |
| Toyama | 99.6 | 118.8 | 100.6 |
| Ishikawa | 102 | 91.0 | 103.5 |
| Fukui | 74.2 | 93.6 | 101 |
| Yamanashi | 102.4 | 84.9 | 85.5 |
| Nagano | 163 | 138.6 | 147.6 |
| Gifu | 73.8 | 96.6 | 85.7 |
| Shizuoka | 72.3 | 73.5 | 67.7 |
| Aichi | 91.3 | 107.9 | 100.3 |
| Mie | 113.9 | 107.4 | 109.1 |
| Shiga | 100.4 | 123.6 | 117.6 |
| Kyoto | 120.3 | 114.0 | 122.7 |
| Osaka | 92.7 | 88.2 | 101.3 |
| Hyogo | 104.4 | 100.6 | 122.2 |
| Nara | 45.8 | 41.3 | 48.4 |
| Wakayama | 139 | 134.9 | 119.5 |
| Tottori | 161.7 | 133.0 | 151.7 |
| Shimane | 123.1 | 113.3 | 142.1 |
| Okayama | 61.2 | 53.5 | 68.9 |

| **Prefecture** | **SCR** | | |
| --- | --- | --- | --- |
|  | **2016** | **2017** | **2018** |
| Hiroshima | 92.7 | 95.0 | 105.9 |
| Yamaguchi | 99 | 95.4 | 103.4 |
| Tokushima | 118.1 | 137.3 | 133.4 |
| Kagawa | 83.5 | 113.0 | 124.4 |
| Ehime | 79 | 72.0 | 81.2 |
| Kochi | 86 | 120.2 | 123.9 |
| Fukuoka | 93.4 | 101.0 | 104.3 |
| Saga | 130.1 | 113.4 | 104.7 |
| Nagasaki | 101.2 | 110.1 | 96.6 |
| Kumamoto | 69.7 | 68.1 | 73.9 |
| Oita | 100.1 | 131.2 | 113.8 |
| Miyazaki | 94.8 | 112.1 | 100.7 |
| Kagoshima | 93.3 | 99.4 | 109.1 |
| Okinawa | 94.9 | 104.3 | 102.7 |

SCR: standardized claim ratio
